# The male germ cell gene regulator CTCFL is functionally different from CTCF and binds CTCF-like consensus sites in a nucleosome composition-dependent manner

**DOI:** 10.1186/1756-8935-5-8

**Published:** 2012-06-18

**Authors:** Frank Sleutels, Widia Soochit, Marek Bartkuhn, Helen Heath, Sven Dienstbach, Philipp Bergmaier, Vedran Franke, Manuel Rosa-Garrido, Suzanne van de Nobelen, Lisa Caesar, Michael van der Reijden, Jan Christian Bryne, Wilfred van IJcken, J Anton Grootegoed, M Dolores Delgado, Boris Lenhard, Rainer Renkawitz, Frank Grosveld, Niels Galjart

**Affiliations:** 1 Department of Cell Biology Erasmus Medical Center, Rotterdam, The Netherlands; 2 Institut für Genetik, Justus-Liebig-Universität, Giessen, Heinrich-Buff-Ring 58-62, 35392, Giessen, Germany; 3 Computational Biology Unit, Bergen Center for Computational Science, University of Bergen, Thormøhlensgate 55, N-5008, Bergen, Norway; 4 Department of Molecular Biology, Instituto de Biomedicina y Biotecnología de Cantabria, IBBTEC Universidad de Cantabria-CSIC-IDICAN, Santander, Spain; 5 Cell Cycle, Stem Cell Fate and Cancer Laboratory, Fundación Marqués de Valdecilla (IFIMAV), Santander, Spain; 6 Center for Biomics Erasmus Medical Center, Rotterdam, The Netherlands; 7 Department of Reproduction and Development Erasmus Medical Center, Rotterdam, The Netherlands; 8 Cancer Genomics Center, NGI, Rotterdam, The Netherlands; 9 Center for Biomedical Genetics, Rotterdam, The Netherlands

**Keywords:** CTCF, CTCFL, Gametogenesis, Genome-wide binding, Nucleosome

## Abstract

**Background:**

CTCF is a highly conserved and essential zinc finger protein expressed in virtually all cell types. In conjunction with cohesin, it organizes chromatin into loops, thereby regulating gene expression and epigenetic events. The function of CTCFL or BORIS, the testis-specific paralog of CTCF, is less clear.

**Results:**

Using immunohistochemistry on testis sections and fluorescence-based microscopy on intact live seminiferous tubules, we show that CTCFL is only transiently present during spermatogenesis, prior to the onset of meiosis, when the protein co-localizes in nuclei with ubiquitously expressed CTCF. CTCFL distribution overlaps completely with that of Stra8, a retinoic acid-inducible protein essential for the propagation of meiosis. We find that absence of CTCFL in mice causes sub-fertility because of a partially penetrant testicular atrophy. CTCFL deficiency affects the expression of a number of testis-specific genes, including Gal3st1 and Prss50. Combined, these data indicate that CTCFL has a unique role in spermatogenesis. Genome-wide RNA expression studies in ES cells expressing a V5- and GFP-tagged form of CTCFL show that genes that are downregulated in CTCFL-deficient testis are upregulated in ES cells. These data indicate that CTCFL is a male germ cell gene regulator. Furthermore, genome-wide DNA-binding analysis shows that CTCFL binds a consensus sequence that is very similar to that of CTCF. However, only ~3,700 out of the ~5,700 CTCFL- and ~31,000 CTCF-binding sites overlap. CTCFL binds promoters with loosely assembled nucleosomes, whereas CTCF favors consensus sites surrounded by phased nucleosomes. Finally, an ES cell-based rescue assay shows that CTCFL is functionally different from CTCF.

**Conclusions:**

Our data suggest that nucleosome composition specifies the genome-wide binding of CTCFL and CTCF. We propose that the transient expression of CTCFL in spermatogonia and preleptotene spermatocytes serves to occupy a subset of promoters and maintain the expression of male germ cell genes.

## Background

Three-dimensional folding of the eukaryotic genome occurs in a highly organized manner so as to compact chromatin while allowing temporal and spatial expression of genes. The genome contains regulatory elements, such as promoters, enhancers, locus control regions, insulators and enhancer blockers, that can orchestrate chromatin folding and gene activity over short and long distances, both in cis and in trans [1]. CTCF is a key coordinator of three-dimensional chromatin structure, allowing loop formation and specific chromatin compositions [2,3]. Gene activity is controlled in a positive or negative manner depending on the regulatory sequences present in the loops that are formed. The importance of CTCF in chromatin organization is emphasized by its evolutionary conservation, its ubiquitous expression, and its essential role in virtually all cells and tissues examined [4,5]. Hence, CTCF has been termed the “master weaver” of the genome [3].

The genome-wide binding by CTCF has been studied by different groups (see, for example, [6-10]). This has revealed ~35,000 CTCF-binding sites in the mammalian genome, of which more than 70% are shared between cell types. A relatively long consensus-binding motif for CTCF has been determined, which displays variability when compared to sites of transcription factors like KLF4, SOX2 and MYC [7]. The majority of CTCF binding-sites are found near genes, and ~8% is in the vicinity of transcription start sites (TSSs). Arrays of positioned (or “phased’) nucleosomes are found surrounding the nucleosome-free CTCF-binding sites [11-13], suggesting that CTCF binding promotes the ordered positioning of histones in its vicinity. CTCF has also been proposed to regulate the positioning of variant histones, such as H2A.Z [6,14]. Interestingly, the cohesin complex binds at the same position as CTCF in a CTCF-dependent manner. Together with CTCF, cohesin is essential for a proper three-dimensional chromatin structure and correct gene regulation [15-17].

CTCF-dependent loop formation is of crucial importance at imprinted loci. A well-studied example is the imprinted *Igf2-H19* locus, in which *Igf2* is expressed from the paternal and *H19* from the maternal allele [18]. The imprinting control region (ICR) located in between the *Igf2* and *H19* genes is methylated on the paternal allele, preventing CTCF binding. As a result the enhancer downstream of the *H19* gene can interact with the *Igf2* promoter and drive expression of this gene. On the non-methylated maternally derived ICR, CTCF does bind, thereby preventing enhancer-*Igf2* interaction, resulting in a chromatin loop that allows enhancer-*H19* association and *H19* expression. By binding the ICR, CTCF therefore acts as a regulator of imprinted sites.

The CTCF-like (CTCFL) protein, or Brother Of the Regulator of Imprinted Sites (BORIS) [19], has a central domain of 11 zinc fingers (ZFs) that is very similar to that of CTCF and that is essential for DNA binding. The N- and C-terminal domains of CTCF and CTCFL are not homologous. CTCFL is less conserved across species, and the protein arose later in evolution, as it is detected in amniotes only [20]. Furthermore, expression of CTCFL is restricted to testis, several types of cancers and a number of cell lines [21-23].

Studies of CTCF and CTCFL protein distribution in the testis have yielded contradictory results. Initially, a mutually exclusive expression pattern of CTCFL and CTCF was described [19], with CTCF being present in round spermatids (i.e. after meiosis) and CTCFL in primary spermatocytes (i.e. during meiotic prophase).

Surprisingly, CTCFL was reported to be more abundant in the spermatocyte cytoplasm than in the nucleus. This led to the hypothesis that during germ cell development, CTCFL substitutes for the absence of CTCF and might be involved in reprogramming of DNA methylation in the male germ line. CTCFL was later reported to be present in gonocytes during embryonic development and, after birth, in spermatogonia, whereas CTCF was reported to localize to the supporting Sertoli cells [24]. In the same study CTCFL, together with the protein methyltransferase PRMT7, was suggested to regulate DNA methylation of imprinted genes in the male germline. However, defects in imprinting often result in embryonic phenotypes [25], whereas Ctcfl knockout mice were shown to display a phenotype only in the testis [26]. Recently, enrichment of *Ctcfl* mRNA in round spermatids was reported, adding perplexity to the localization and expression of CTCFL [26,27].

While the whole genome DNA-binding profile for CTCF has been elucidated, this has not been done for CTCFL. It therefore remains unclear how CTCFL binding relates to that of CTCF. In addition, it is unknown how these proteins are related functionally and mechanistically. To address these issues, we examined CTCFL function and localization with respect to CTCF, and identified the genome-wide binding sites of CTCFL and CTCF. We show that CTCF and CTCFL are functionally different proteins that co-localize within the nuclei of pre-meiotic germ cells. CTCFL acts as a male germ cell gene regulator, preferably binding near promoters with active chromatin marks. Interestingly, CTCF and CTCFL bind a highly similar DNA motif; nevertheless, only two-third of the ~5,700 CTCFL-binding sites are bound by CTCF. Conversely, the vast majority of CTCF sites are not bound by CTCFL. We find that nucleosome composition specifies CTCF and CTCFL binding. In contrast to CTCF, CTCFL associates with relatively “open” chromatin, and we propose that CTCFL promotes the maintenance of the epigenetic state of a subset of gene promoters and hence gene expression during spermatogenesis.

## Results

### CTCFL and CTCF co-localize transiently in pre-meiotic male germ cells

**Figure 1:**
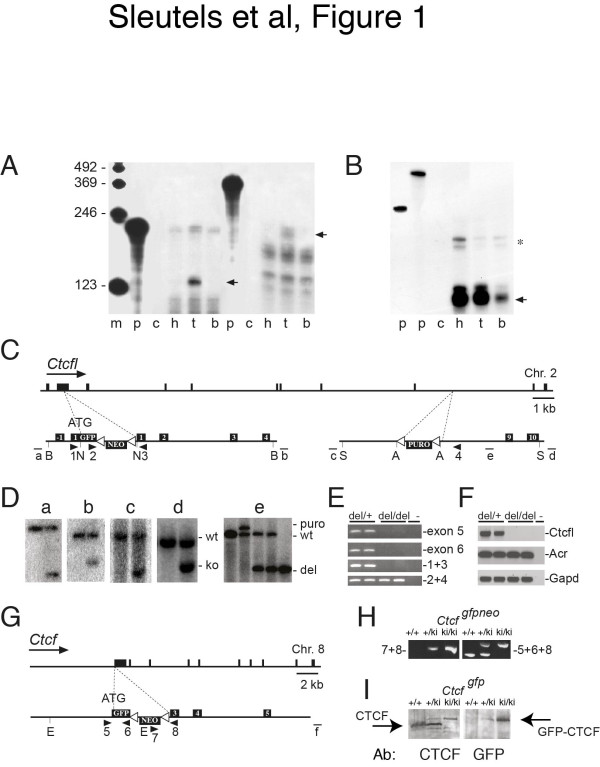
**Ctcfl and Ctcf expression and targeting. AB** RNAse protection analysis of Ctcfl and Ctcf. For Ctcfl (**A**) RNase protection analysis (RPA) was performed on polyA purified mRNA with probes covering parts of Ctcfl exon 8 and 9 (*left*, small fragment) or a 5’end RACE product (*right*, large fragment). For Ctcf (**B**) the RPA was performed on total RNA with probes protecting Ctcf exon 2. The positions of the respective protected fragments are indicated with *arrows*. *Ctcfl* mRNA mRNA can only be detected in polyA purified mRNA from testis (*t*), whereas Ctcf is identified in total RNA from all three tissues tested. *M*, marker; *p*, input probe; *c*, tRNA control; *h*, heart; *t*, testis; *b*, brain. Aprt exon 3 is used as loading control and marked by an *asterisk*[28]. This analysis identifies the first exon containing the ATG translation initiation codon in Ctcfl and shows that Ctcfl is expressed in testis. **C** Schematic overview of the modified Ctcfl alleles and targeting constructs. The Ctcfl locus is shown on scale, with the constructs (not on scale) used for homologous recombination in ES cells underneath. Targeting at the 5’end of Ctcfl yielded the *Ctcfl*^*gpf- neo*^ allele. Cre-mediated excision of the LoxP-embedded neomcyin resistance gene yielded the *Ctcfl*^*gfp*^ allele (not shown). The 3’end targeting was performed on the *Ctcfl*^*gpf- neo*^ allele, and yielded the *Ctcfl*^*gfp*-neo-puro^ allele. Cre-mediated excision of the sequence in between the outermost LoxP sites yielded the *Ctcfl*^*del*^ allele, in which exons 1–8 of the Ctcfl gene are deleted (not shown). A major difference between the *Ctcfl*^*del*^ allele described here and the Ctcfl knockout published earlier [26] is that in the *Ctcfl*^*del*^ allele the GFP coding sequence is fused in frame with the CTCFL coding sequence. *Black boxes* represent exons, GFP tag, neomycin and puromycin cassettes. Probes a, b, c, d and e are indicated by *lines*. Oligos 1, 2, 3 and 4 are represented by *arrowheads*. *White triangles* are LoxP sites. *B* = BglII; *N* = NcoI; *S* = SpeI; *A* = AvrII. **D** DNA blot showing Ctcfl targeting. Probes a and b were used on DNA blots from ES cells for identification of the *Ctcfl*^*gfp*-neo^ allele and probes c and d for the Ctcfl^puro^ allele. Probe e identifies the *Ctcfl*^*del*^ allele from *Ctcfl*^*gfp*-neo-puro^ mice that were crossed to a chicken Actin-Cre transgene. Probe a, HindIII digest (wt 8.9 kb, ko 5.7 kb); probe b, EcoRI digest (wt 14 kb; ko 11 kb); probe c, BamHI digest (wt 16.1 kb; ko 6.8 kb); probe d, BamHI digest (wt 16.1 kb; ko 11.1 kb). **E** Absence of Ctcfl DNA in the *Ctcfl*^*del*^ allele. PCR on tail DNA indicates that *Ctcfl*^*del/del*^ mice are deleted for exons 1–8 (*top three panels*) and are positive for GFP (oligos 2 and 4). **F** Absence of Ctcfl RNA in Ctcfl mutant mice. PCR on cDNA derived from testis mRNA shows that Ctcfl is absent from *Ctcfl*^*del/del*^ mice. Acrosin and Gapd function as positive controls. **G** Schematic overview of the Ctcf allele and targeting strategy for the Ctcf^gfp-neo^ allele. The Ctcf locus is shown on scale, with the construct (not on scale) used for homologous recombination in ES cells underneath. Cre-mediated excision of the LoxP-embedded neomcyin resistance gene yielded the Ctcf^gfp^ (or Ctcf^ki^) allele (not shown). *Black boxes* represent exons, GFP tag and neomycin cassette. Oligos 5, 6, 7 and 8 are represented by *arrowheads*. *White triangles* are LoxP sites. *E* = EcoRI. **H** PCR confirming Ctcf^gpf-neo^ allele. Identification of the CTCF^gfp-neo^ (or Ctcf^ki^) allele by PCR with oligos 7 and 8 or oligos 5, 6 and 8 (see panel **G**). **I** Western blot confirming GFP-CTCF expression from the Ctcf^gfp^ allele. We isolated MEFS from E13.5 day wild-type (+/+), heterozygous Ctcf^gfp/+^ (or Ctcf^ki/+^) or homozygous Ctcf^gfp/gfp^ (or Ctcf^ki/ki^) embryos, and identified the GFP-CTCF fusion protein by Western blot of MEF extracts using anti-CTCF or anti-GFP antibodies. Note the increased size of the GFP-CTCF protein compared to the CTCF protein due to the GFP tag.

To resolve the localization of CTCF and CTCFL in testis, and to address CTCFL function, we generated *Ctcfl* knockout and GFP-CTCF- and GFP-CTCFL-expressing knockin mice. To obtain information about the organization of the *Ctcfl* gene, we mapped its 5’ end and examined *Ctcfl* expression (Figure 1A, B). We next generated three separate alleles using homologous recombination in ES cells: a *Ctcfl* knockout allele (*Ctcfl*^*del*^), in which exons 1–8 of the Ctcfl gene are deleted, and Ctcfl and Ctcf knockin alleles (*Ctcfl*^*gfp*^ and Ctcf^gfp^, respectively), to express GFP-CTCFL and GFP-CTCF instead of CTCFL and CTCF, respectively (Figure 1C-I).

**Figure 2:**
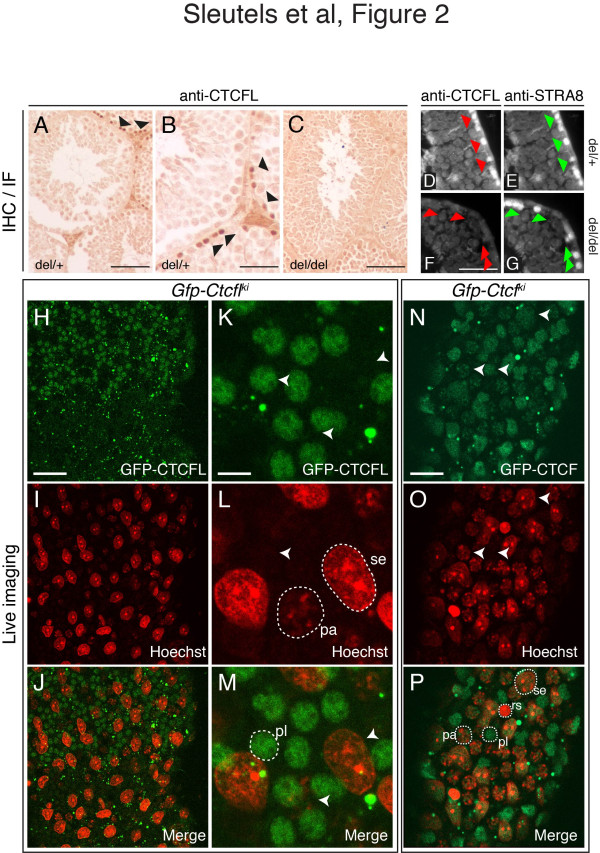
**Expression of CTCFL and CTCF in the testis. A**-**C** Immunohistochemical staining of testis sections. Paraffin-embedded sections from day 90 testes from heterozygous (del/+) and homozygous (del/del) Ctcfl mutant mice were stained with anti-CTCFL, followed by diaminobenzidine (*DAB*) coloring. Some of the CTCFL-positive cells are indicated with *black arrowheads*. Scale bars **A**, **C**: 100 μm, **B**: 50 μm. **D**-**G** Immunofluorescence staining of testis sections. Sections as described in **A**-**C** were stained with CTCFL (**D** and **F**) or STRA8 (**E** and **G**) antibodies. STRA8-positive cells in panels **E** and **G** are indicated with *green arrowheads*; the same cells are indicated with *red arrowheads* in the sections stained with anti-CTCFL antibodies (panels **D** and **F**). In Ctcfl mutant mice, STRA8 distribution is not changed. Scale bar is 50 μm. **H**-**P** Ex vivo confocal and multiphoton imaging of intact seminiferous tubules. Testis tubules were dissected from GFP-CTCFL- (**H**-**M**) or GFP-CTCF- (**N**-**P**) expressing mice, exposed to Hoechst at the adluminal side of the seminiferous tubule, and analyzed with a confocal/multiphoton microscope (GFP-CTCFL and GFP-CTCF, *green*; Hoechst, *red*). Panel **H**-**J** (see also Movie S1) shows a low magnification view of GFP-CTCFL distribution. Notice the presence of GFP-CTCFL-positive cells in the upper half of the tubule and their absence in the bottom half, indicating a transient population of cells. In (**K**-**M**) a high-magnification view of the same GFP-CTCFL-positive cells is shown. Notice the non-homogenous distribution of GFP-CTCFL in the nucleus. In (**N**-**P**) GFP-CTCF staining is shown. For clarity, some of the cell types are encircled, and their position is indicated in the other panels using *white arrowheads*. *Pl* = preleptone spermatocyte; *rs* = round spermatid; *pa* = pachytene spermatocyte; *se* = Sertoli cell. Bars, **H**-**J**: 70 μm, **K**-**M**: 10 μm, **N**-**P**: 25 μm.

Mice were generated, and the distribution of CTCFL was investigated by immunocytochemistry in sections of seminiferous tubules from wild-type and Ctcfl knockout mice. CTCFL was present in wild-type testis in cells lining the basal lamina (Figure 2A, B). Not all cells lining the lamina were CTCFL-positive, and in some tubules no CTCFL-positive cells were detected. Importantly, no signal was detected on sections derived from CTCFL-deficient mice (Figure 2C, see also Figure 2F), showing that the

CTCFL staining in wild-type sections is specific. The localization of the CTCFL-positive cells in the basal compartment of the seminiferous tubules indicates that these cells are spermatogonia or preleptotene spermatocytes, as only upon progression in meiotic prophase do spermatocytes become disconnected from the basal lamina and move through the Sertoli cell barrier into the adluminal compartment of the seminiferous tubules.

The localization of CTCFL appeared reminiscent of STRA8 (STimulated by Retinoic Acid), which is expressed transiently from B spermatogonia to preleptotene spermatocytes and is essential for retinoic acid-induced commitment to meiosis [29-32]. An absolute overlap between STRA8 and CTCFL was confirmed using dual-color immunofluorescence (Figure 2D, E). Immunofluorescent staining experiments did not reveal an obvious change in the number of STRA8-positive tubules in CTCFL-deficient testis (Figure 2E, G, and data not shown). Thus, absence of the CTCFL signal in Ctcfl knockout sections is not due to the disappearance of a cell type.

To confirm CTCFL localization and compare its distribution to that of CTCF, we next analyzed expression and localization of the two proteins ex vivo. We isolated intact seminiferous tubules from the testes of *Ctcfl*^*gfp*^ and Ctcf^gfp^ male mice, which were injected with Hoechst via the rete testis to stain nuclei of cells at the adluminal compartment of the tubule. We then visualized GFP-CTCF(L) and Hoechst concomitantly using a multiphoton confocal laser scanning microscope setup [33]. Three-dimensional reconstruction of images taken longitudinally through the seminiferous tubules yielded an organizational view of the tubule, and the position and type of the GFP-positive cells (Figure 2H-P). GFP-CTCFL was detected in the nucleus of clustered cells representing a minor fraction of the total testis tubule (Figure 2H-m, and Additional file 1: Movie S1). These cells stained negative for Hoechst, and since the luminally injected Hoechst does not cross the Sertoli cell barrier, the GFP-CTCFL-positive cells must reside on the basal side of this barrier. Sertoli cells, which form the tight junctions of the Sertoli cell barrier, were Hoechst-positive (Figure 2I, J, L, M and Additional file 1: Movie S1). Primary spermatocytes pass this barrier in the preleptotene and leptotene stage [34]. Based on Hoechst staining, morphology, size and location, we conclude that the GFP-CTCFL positive cells represent spermatogonia and preleptotene spermatocytes. The ex vivo GFP/Hoechst results obtained in live tissue are consistent with our data obtained in fixed paraffin-embedded sections of the testis stained with the CTCFL antibodies (Figure 2A-C). Together with the STRA8 colocalization data, they strongly suggest that in the adult testis CTCFL is transiently expressed in late spermatogonia and preleptotene germ cells.

In contrast to GFP-CTCFL, GFP-CTCF was present in the nucleus of all cell types of the seminiferous tubule, including all germ cells prior to spermiogenesis (Figure 2N-P, and data not shown). GFP-CTCF was also expressed in round spermatids, albeit at lower levels. This is consistent with a primary role for CTCF in cells with histone-based chromatin. Thus, live imaging in seminiferous tubules shows that CTCF and CTCFL are co-expressed within late spermatogonia and preleptotene spermatocytes. Measurement of GFP fluorescence intensities indicate that in cells where both proteins are expressed, the level of CTCF is somewhat higher than that of CTCFL.

### CTCFL is important for spermatogenesis

To study the role of CTCFL in the male germ line, we analyzed *Ctcfl*^*del*/+^ and *Ctcfl*^*del/del*^ mice. These mice demonstrated no gross phenotypic defects and appeared normal. Heterozygous and homozygous *Ctcfl*^*del*^ females showed normal fertility and yielded offspring with expected ratios (data not shown), consistent with a role for CTCFL in spermatogenesis only. Heterozygous *Ctcfl*^*del*/+^ males generated offspring, and demonstrated normal fertility (Table 1). However, homozygous *Ctcfl*^*del*^ male littermates generated offspring in only half (14 out of 27) of the breedings (Table 1). Breeding with *Ctcfl*^*del/del*^ males yielded significantly (*p* = 0.01; chi test) fewer litters than *Ctcfl*^*del*/+^ males, but not a different litter size (*p* = 0.11; *t*-test). These data indicate that CTCFL is important for male fertility.

**Table 1:** Sub-fertility of CTCFL mutant mice

**Genotype**	***Ctcfl***^***del*****/+**^	***Ctcfl***^***del/del***^
Number (percentage) of breedings w/o offspring	3/60 (5%)	14/27 (51.9%)
Average number of offspring per litter (± SD)	7.7 ± 2.6	6.4 ± 2.8

**Figure 3:**
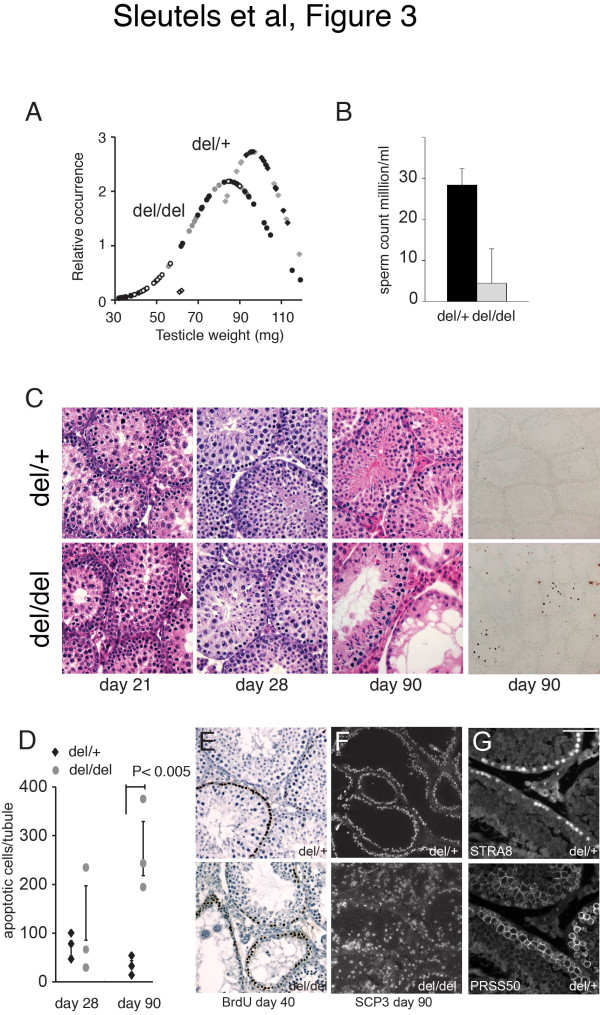
**CTCFL is important for spermatogenesis. A** Testicular weight distribution. The testicular weight of Ctcfl heterozygous (*Ctcfl*^*del*/+^; *diamonds*) and homozygous (*Ctcfl*^*del/del*^; *circles*) mice was measured and plotted as a normalized probability distribution (i.e., the surface under the curve represents a total probability of 1). Testes of knockout mice are significantly smaller (*p* < 0.0005, *t*-test). *White symbols* represent infertile males, *black symbols* are fertile males, and *grey symbols* correspond to males not tested for fertility. **B** Ctcfl mutant mice display reduced fertility. Epididymal sperm count from Ctcfl heterozygous (*Ctcfl*^*del*/+^; *black bar*) and homozygous (*Ctcfl*^*del/del*^; *grey bar*) mice. Standard deviation is plotted (*p* = 0.0002, *n* = 4). **C** Testis histology. In the *left three panels* a timed series of HE-stained testicle sections is shown (postnatal day 21, 28, 90), while in the *right hand panel* an apoptosis assay (TUNEL staining) of testicle sections at day 90 is shown. Note that in CTCFL-deficient testes some seminiferous tubules appear normal, whereas others (which can be adjacent to the normal ones) have lost most of their meiotic and post-meiotic germ cells, leaving only mitotic spermatogonia (that stain positive for BrdU incorporation, see panel **E**) and Sertoli cells. Yet other tubules contain disorganized spermatocytes, and some of them even elongated sperm. Thus, there is no absolute block in differentiation or progression of germ cell development, but the incomplete penetrance of the infertility phenotype is however directly linked to the testicle weight (panel **A**) and to the degenerative level of the seminiferous tubules. **D** Apoptosis plot. Number of TUNEL-positive apoptotic cells per 100 seminiferous tubules counted at day 28 and day 90. Standard deviation of three animals per genotype and time point is indicated. **E** DNA synthesis marked by a 1-h pulse of BrdU in day 40 testicles reveals that mitotic spermatogonia are still present in degenerated tubules. Counterstaining with hematoxylin. **F** SCP3 staining in spermatocytes of day 90 testicles as a marker for tubule organization. **G** PRSS50 co-localizes only partially with STRA8. Immunofluorescence staining with a STRA8 antibody (*top panel*) or PRSS50 antibody (*bottom panel*) of adult testicle sections shows that PRSS50 and STRA8 expression overlaps only partially. Scale bars are 50 μm.

To further investigate the CTCFL deficiency, we weighed testes from 90-day-old *Ctcfl*^*del/del*^ and *Ctcfl*^*del*/+^ mice and plotted weight distributions. We found that, on average, the CTCFL-deficient testes weighed significantly less compared to testes from heterozygous littermates (Figure 3A). In addition, we found that lower testes weights coincided with infertile males (Figure 3A). The weight distribution shows that there are also normal testes in the *Ctcfl*^*del/del*^ population. Still, on average, the epididymides from homozygous *Ctcfl*^*del*^ mice contained only 15% of sperm compared to heterozygous littermates (Figure 3B).

From day 28 onwards Ctcfl mutant mice displayed loss of germ cells by apoptosis and an increasing level of atrophy that increased with age (Figure 3C, D). Mitotic spermatogonia, staining positive for BrdU incorporation, were still often observed (Figure 3E), whereas SCP3, a marker for spermatocytes, revealed severe tubule disorganization (Figure 3F). In fact, the level of atrophy and disorganization between individual mice and between individual seminiferous tubules within mice was variable, and normal seminiferous tubules could even be adjacent to abnormal ones. Thus, the penetrance of the atrophic testes and sterility phenotype in CTCFL deficiency is incomplete and differs considerably per mouse.

**Figure 4:**
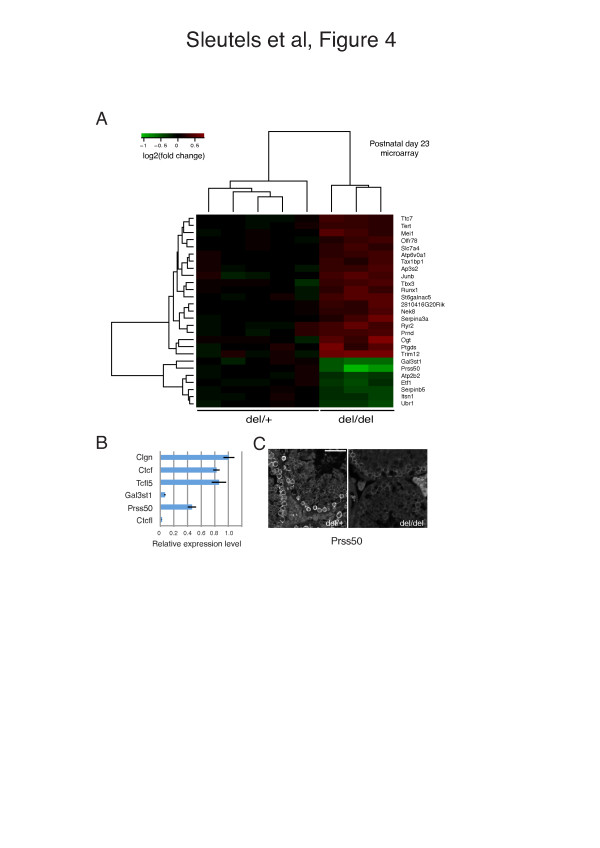
**Regulation of testis-specific genes by CTCFL. A** Heatmap representation of microarray data. We compared five samples from heterozygous and three samples from homozygous Ctcfl knockout mice. Depicted are the top 27 deregulated genes, where the log2-transformed fold change compared to the average expression in heterozygous testis is shown. **B** Expression analyses in Ctcfl mutant testes. Real-time RTPCR expression analyses on testis RNA from Ctcfl mutant mice relative to wild type using Ccna1 as reference. Genes were examined in 90-day-old testes. **C** Reduced PRSS50 expression in Ctcfl mutant testis. Immunofluorescence analysis of testis sections from heterozygous (del/+) and homozygous (del/del) Ctcfl knockout mice, using antibodies against PRSS50.

Next we performed a microarray analysis on day 23 testis mRNA (a time point that precedes the start of apoptosis and degeneration in the testes of CTCFL-deficient mice) on *Ctcfl*^*del/del*^ and *Ctcfl*^*del*/+^ littermates. This revealed several affected genes in *Ctcfl*^*del/del*^ testes (Figure 4A). The Prss50 and Gal3st1 genes were most downregulated (~1.5 fold), which matches results from another study [26]. Real-time RT-PCR verified results from the microarray (Figure 4B).

GAL3ST1 is crucial for spermatogenesis as mutant mice are infertile because of an arrest at the end of the meiotic prophase [35]. PRSS50 (Testis Specific Protease) has an exclusively testicular expression pattern, and is detected both in CTCFL-positive cells as well as in later stages of spermatogenesis [36], including STRA8/CTCFL-negative pachytene spermatocytes (Figure 3v). Since a CTCFL-deficiency affects differentiation of cells subsequent to the preleptotene stage, the reduction in Prss50 and Gal3st1 mRNA may be the result of a reduction in the number of cells going through meiosis or a reduction in Prss50 and Gal3st1 mRNA per cell. We therefore investigated PRSS50 expression in sections of wild-type and CTCFL-mutant mice and noted reduced protein levels per cell (Figure 4C).

### CTCFL regulates testis-specific gene expression

**Figure 5:**
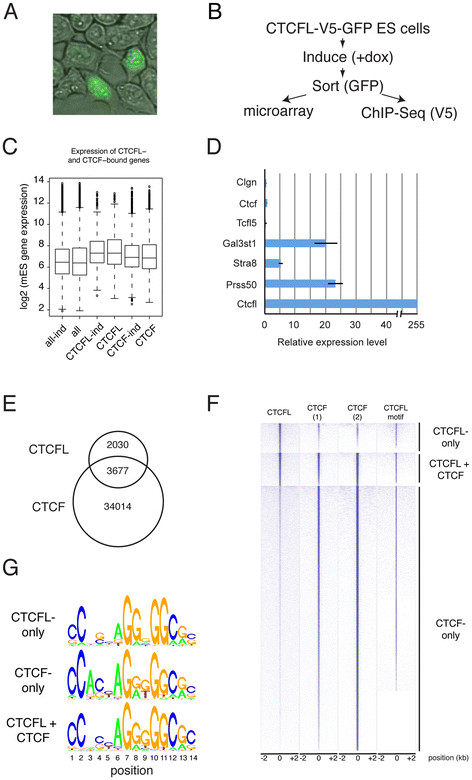
**Genome-wide analysis of CTCFL expression in ES cells. A** Inducible expression of CTCFL-V5-GFP in ES cells. Notice the nuclear localization of CTCFL-V5-GFP in cells expressing the protein. **B** Flow chart of experiments. ES cells with a Tet-on inducible expression of a CTCFLV5-GFP transgene were sorted for GFP and used for microarray and ChIP-Seq analyses. **C** CTCFL expression and DNA binding are associated with elevated gene expression levels. We plotted gene expression levels, as determined by microarray analysis of induced (*ind*) or non-induced ES cells, for all genes (*all*), or those bound by CTCF, or CTCFL, to the respective promoter region (−2 k to +1 kb around TSS). Differences are highly significant (*p*-value CTCF-ind versus CTCFL-ind: 5.1 × e^-14^; *p*-value CTCF versus CTCFL: 5.9 × e^-13^). **D** Transcript analyses in ES cells expressing CTCFL-V5-GFP. Real-time RT-PCR expression analyses of CTCFL-V5-GFP-induced and GFP-sorted ES cells, relative to non-induced ES cells, for the indicated genes, referenced to Cdk2 expression. **E** Venn diagram of DNA-binding sites for CTCFL and CTCF. **F** Clustered heatmap representation of three classes of CTCF/CTCFL-binding sites. Shown are the binding profiles of CTCFL and CTCF (*1*: our own data; *2*: [7]) across all CTCF/CTCFL-binding sites identified in mES cells. Sites are grouped into CTCFL-only, CTCF-only, and combined CTCFL and CTCF sites. Within the three classes, data sets were sorted decreasingly from *top* to *bottom* for average binding across the interval from 2 kb to +2 kb around the identified binding peak *center* positions. Additionally the occurrences of predicted CTCFL motifs within these intervals are plotted. **G** Motif comparison of CTCF and CTCFL. DNA-binding motif for CTCFL-only (*top panel*), CTCF + CTCFL (*middle panel*) and CTCF-only binding sites (*bottom panel*).

The whole genome DNA-binding profile for CTCF has been elucidated in several cell systems (see, for example, [6-9]). We sought to compare the DNA-binding profiles for CTCF and CTCFL in the same cell type. Since CTCF is ubiquitously expressed in the testis, whereas the presence of CTCFL is highly restricted, genome-wide DNA-binding patterns derived from whole or partially purified testis preparations cannot be compared (see Discussion). We therefore generated ES cells, a cell type closely related to germ cells [37-40], in which expression of a V5- and GFP-tagged CTCFL protein could be induced (Figure 5A), thereby mimicking the CTCFL-positive germ cells that express both CTCFL and CTCF. Advantages of this system are furthermore the unlimited source of cells and the possibility to sort for GFP-positive cells that express the fusion protein to obtain a pure population of cells. In addition, the V5 tag permits stringent and exclusive immunoprecipitation of CTCFL. Thus, with this system genome-wide RNA (micro-arrays) and DNA-binding studies (ChIP-Seq) were carried out (Figures 5B).

Comparison of the expression of all genes on the microarray to expression of genes bound by CTCF or CTCFL revealed that the CTCFL-bound genes were, on average, more abundantly expressed (Figure 5C). The same held true when CTCFL-bound genes were compared to random gene sets (not shown). These data indicate that CTCFL associates with active genes. Several genes upregulated in CTCFL-GFP-V5-induced ES cells were also detected in the list of genes downregulated in CTCFL-deficient testes. Real-time RT-PCR confirmed that Gal3st1, Prss50 and even Stra8 expression were upregulated in CTCFL-induced ES cells (Figure 5D). Thus, CTCFL can act on male-specific germ cell genes in ES cells, and two of the most downregulated genes in CTCFL-deficient testis are upregulated in CTCFL-GFP-V5-expressing ES cells. These data underscore the notion that ES cells resemble germ cells and indicate that CTCFL acts as a male germ cell gene regulator.

### Genome-wide binding of CTCFL and CTCF

To determine the genome-wide binding pattern of CTCFL, we used GFP-sorted CTCFLV5-GFP-induced ES cells, which express both CTCFL and CTCF. The V5 antibody was used for ChIP of CTCFL, with non-induced ES cells as control. Normal ES cells and a rabbit polyclonal antibody to CTCF [4] were used for ChIP-sequencing of CTCF. ChIP- sequencing revealed 5707 CTCFL and 37691 CTCF-binding sites (Figure 5E). To validate our data, we compared the number and position of CTCF sites determined by us with published data from the same cell type [7] and found a very high overlap (Figure 5F). Sorting the CTCFL-binding sites on the number of unique sequence reads yielded a list of genes that was headed by Stra8 and Prss50 (Table 2), two genes that are upregulated in CTCFL-inducible ES cells. Thus, the most prominent CTCFL sites locate at genes that are important for germ cells.

**Table 2:** Top ten CTCFL-binding sites in induced ES cells

	**Chr**	**Position**	**Gene**	CTCFL bound*	**CTCF bound**
1	6	34,872,000	Stra8	134	Yes
2	9	110,760,000	Tsp50	113	Yes
3	14	103,450,500	Irg1	105	Yes
4	9	106,114,000	Twf2	101	Yes
5	9	50,260,500	-	100	Yes
6	5	125,061,000	Tctn2	97	Yes
7	8	107,058,500	Nae1	95	Yes
8	2	29,475,000	Rapgef1	94	Yes
9	9	108,838,500	Uqcrc1	93	Yes
10	12	112,970,500	Bag5	92	Yes

*Sites are ranked based on the number of ChIP-sequence reads, filtered for duplicates.

Interestingly, only 64% (3677) of CTCFL sites overlap with those of CTCF; conversely, only ~10% of CTCF sites are bound by CTCFL (Figure 5E). Despite their partial overlap, CTCFL and CTCF bind almost identical consensus sequences (Figure 5G). The most notable differences in the DNA-binding motif are the lower prevalence of a C at positions 1 and 2, the absence of A at position 3 and a lower prevalence for A at position 6, as well as a higher prevalence of G at positions 8 and 11, for the CTCFL motif relative to the one of CTCF. Whether subtle motif differences relate to differences in numbers of binding sites or to effects mediated through CTCFL and/or CTCF are questions currently under investigation. We also noted that, similar to CTCF [6,9,41], not all binding sites for CTCFL contain a consensus motif (Figure 5F).

### Nucleosome occupancy specifies binding of CTCFL versus CTCF

**Figure 6:**
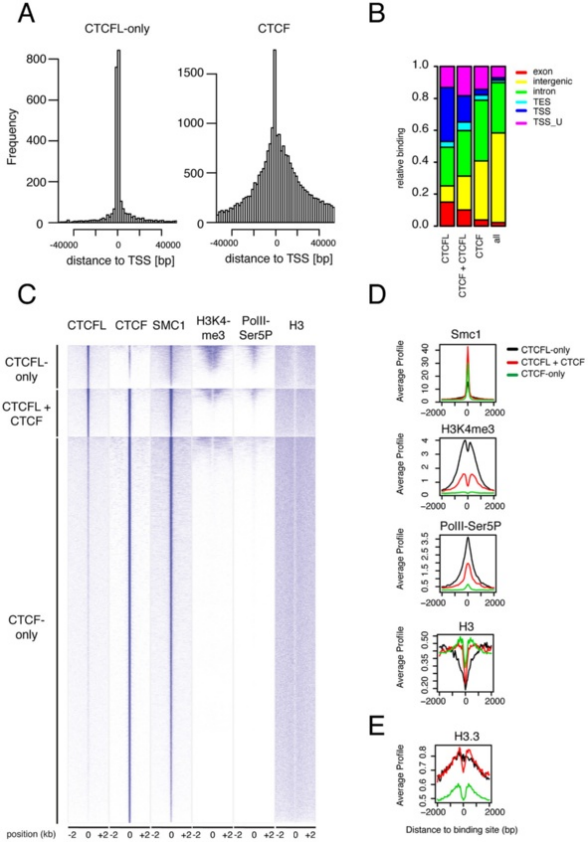
**Characterization of CTCFL and CTCF binding. A** A large fraction of CTCFL-binding sites is located close to promoters. We determined for each CTCFL-only-binding site the distance to the nearest transcriptional start site (TSS) and plotted the frequencies of binding sites in the depicted window from −40 kb to +40 kb around the center of CTCFL-binding sites. CTCF is plotted as comparison. **B** Comparison of the genomic distribution of CTCF- and CTCFL-binding sites. Sites are separated into CTCF-only, CTCFL-only and (CTCF + CTCF). The entire genome is also plotted (*all*). The binding location is separated into exon, intron, intergenic, transcription start site (*TSS*) and transcriptional end sites (*TES*), and plotted as frequencies of total (Y ax). **C** Clustered heatmap representation of the three different classes of CTCF/CTCFL-binding sites with respect to chromatin context. We compared CTCF and CTCFL binding to published ChIP-sequencing data sets for the cohesin complex subunit Smc1, H3K4me3, a phosphorylated form (serine 5) of RNA PolII, (PolIISer5P) and histone H3 [8,42,43]. **D** Cumulative profiles across the three different classes of CTCF/CTCFL-binding sites with respect to chromatin context. The average ChIP-sequencing profiles are shown for the same data sets as in (**C**). **E** Cumulative profiles across the three different classes of CTCF/CTCFL-binding sites with respect to H3.3.

Further analysis of the genome-wide binding of CTCF(L) revealed that CTCFL binds almost exclusively to CTCF consensus sites near promoter areas, in contrast to CTCF (Figure 6A, B). We next split CTCF(L)-binding sites into three groups, i.e., CTCFL-only sites, CTCFL + CTCF sites and CTCF-only sites, and compared CTCF(L) binding to published data sets of transcription factors and other chromatin constituents. Binding sites are shown as heatmaps, which represent individual ChIP-Seq profiles from -2 kb to +2 kb relative to the center (peak maximum) of the analyzed peaks (Figure 6C) and as cumulative profiles (Figure 6D, E), which represent average ChIP-Seq profiles. Sites were sorted for binding strength within the three subsets. This comparative analysis revealed, for example, that CTCFL colocalizes with cohesin at CTCF consensus sites that are not occupied by CTCF (Figure 6C, D). In addition, CTCFL is enriched at transcriptionally active promoters, which are marked by H3K4me3 and PolII phosphorylation on serine 5 (Figure 6C, D). By contrast, CTCF-only sites are not associated with these marks. These data confirm the observation that CTCFL associates with transcriptionally active genes (see Figure 5C).

When we compared binding of CTCF(L) to that of histone H3 we noted that CTCFL preferentially binds large H3-depleted areas (Figure 6C, D). By contrast, CTCF is enriched on sites that display H3 phasing around the CTCF-binding site (Figure 6D). These sites, in turn, do not attract CTCFL. Shared CTCFL/CTCF sites associate with “intermediate” H3-free regions (Figure 6C, D). As the H3-binding site analysis was performed in ES cells that do not express CTCFL, we conclude that the H3 depletion in these cells is not caused by CTCFL, but that H3-depleted regions appear to attract CTCFL.

It has been observed that many “H3-free” regions in the genome in actual fact do contain histones, but that these are loosely assembled and are lost upon DNA extraction with high salt [44]. The variant histone H3.3 has been shown to occupy these areas, often together with another variant histone, H2A.Z [45]. We therefore compared CTCFL binding to that of H3.3 (for which data are available in mouse ES cells [46]) and found that these two proteins colocalize (Figure 6E), whereas CTCF does not associate with H3.3-enriched regions. We conclude that in addition to nucleotide sequence, nucleosome occupancy and composition specify the genome-wide binding of CTCFL and, surprisingly, of CTCF.

### Competition between CTCFL and CTCF on distinct sites

**Figure 7:**
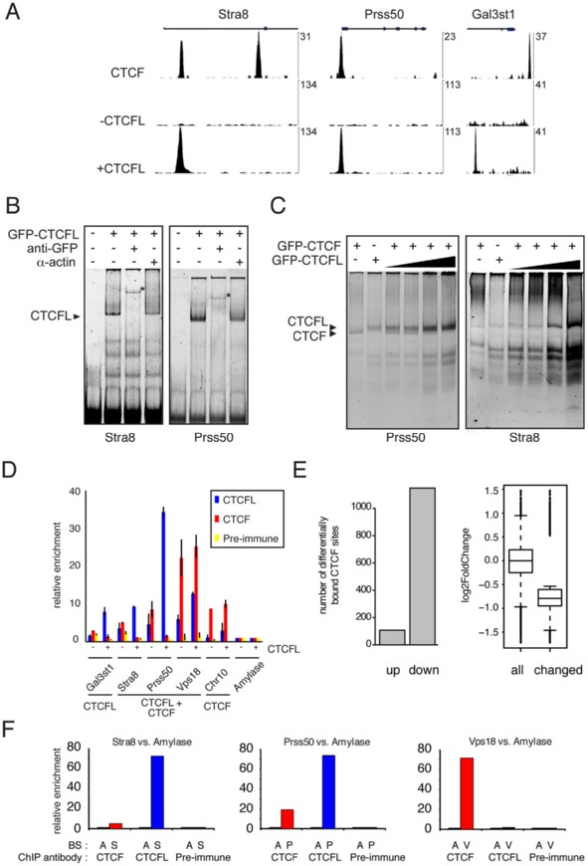
**Characterization of CTCF and CTCFL binding. A** Examples of CTCF- and CTCFL-binding site location. The genomic location of CTCF (*upper part*) and CTCFL (*middle* and *bottom* parts) binding sites in the absence (−CTCFL, *middle*) or presence (+CTCFL, *bottom*) of CTCFL, within the Stra8, Prss50 and Gal3st1 genes. The vertical axes show the number of unique sequence reads. **B** CTCFL binds to Stra8 and Prss50. Band shift analyses of GFP-CTCFL on Stra8 and Prss50 fragments. GFP-CTCFL binding can be super shifted (marked with *asterisks*) with anti-GFP, but not with an Actin antibody. Band shifts were performed under excess probe conditions. **C** In vitro effect of CTCFL on CTCF binding. Band shift analyses with GFP-CTCF and/or GFP-CTCFL on Prss50- and Stra8-bindings sites. GFP-CTCFL is added in increasing amounts (1-, 2-, 5- and 10-fold compared to GFP-CTCF). To allow competition, the band shift was performed under probe-limiting concentrations. **D** Cellular effect of CTCFL on CTCF binding. ChIP analyses with CTCFL (*blue*), CTCF (*red*) and pre-immune (*yellow*) antisera in ES cells that were either non-transfected (−) or transiently transfected CTCFL-V5-GFP (+). According to ChIP-sequencing data, Prss50, Stra8 and Vps18 bind both CTCF and CTCFL, whereas Gal3st1 only binds CTCFL, and Chr10 only binds CTCF. A CTCF- and CTCFL-negative site within the Amylase gene is used as reference and set to 1. *Error bars* represent standard deviations of biological replicates. **E** Competition between CTCF and CTCFL in ES cells. Genome-wide binding of CTCF was compared to that of CTCFL by ChIP-Seq using non-transfected ES cells and ES cells transiently transfected with GFP-CTCFL. The *left hand panel* shows the effect of CTCFL binding on shared CTCFL/CTCF sites that showed >1.5 fold difference in CTCF binding. The effect is categorized into sites with increased (*up*) or decreased (*down*) CTCF binding. The *right hand panel* shows a more general effect of CTCFL binding on CTCF binding. Here, we examined the change in CTCF binding in all shared CTCF(L)-binding sites (*all*) compared to those shared sites that were significantly changed in CTCF binding (*changed*). The effect on CTCF binding is plotted as log2-fold difference. **F** In vivo CTCF(L) binding. ChIP was performed using anti-CTCF (*red*) or anti-CTCFL (*blue*) antibodies, or pre-immune serum, on the indicated sites (*A*: Amylase, *S*: Stra8, *P*: Prss50, *V*: Vps18) in nuclei from dissociated seminiferous tubules, partly purified by elutriation. Relative enrichment is shown compared to Amylase.

ChIP-sequencing and direct ChIP experiments showed that CTCF and CTCFL bind the same site within the Stra8 and Prss50 promoters, but not in the Gal3st1 promoter (Figure 7A). We therefore tested the idea that these two proteins compete for binding on selected sites. Using in vitro band shift assays, we confirmed that CTCFL and CTCF bind the Stra8 and Prss50 promoters (Figure 7v, C). When proteins were added together we did not observe a higher band, indicating that CTCF and CTCFL do not interact to bind a probe simultaneously (Figure 7C). Instead, with increasing amounts of CTCFL, the amount of bound CTCF diminished (Figure 7C), suggesting that CTCFL and CTCF compete for binding sites in vitro.

To examine whether competition occurs in vivo, we used ChIP analysis on CTCFL-induced and -non-induced ES cells using a selected number of sites. In the presence of CTCFL, the amount of bound CTCF was reduced for both Stra8 and Prss50 (Figure 7D). CTCFL induction had no effect on CTCF in the CTCF-only-binding site within the Chr10 locus (Figure 7D). However, for the shared CTCFL/CTCF-binding site at Vps18 we also saw no effect on CTCF binding (Figure 7D). These ChIP results indicate that CTCFL can compete with CTCF, but only at specific sites. To test this hypothesis on a genome-wide level, we transiently transfected GFP-CTCFL into ES cells and examined differences in CTCF binding in ES cells expressing GFP-CTCFL compared to cells not expressing this protein. As shown in Figure 7E (*left panel*), in the presence of CTCFL, binding of CTCF was reduced on ~1,100 sites, whereas binding on ~100 sites was increased. Binding of CTCF to the affected sites was significantly reduced as compared to all CTCF-binding sites (Figure 7E, *right panel*). Among sites displaying reduced CTCF binding were the Prss50 and Stra8 promoters, but not Vps18 and chromosome 10 binding sites (not shown). These results are consistent with the ChIP data (Figure 7D). We conclude that in ES cells, CTCFL and CTCF compete on distinct sites.

To estimate the physiological relevance of our genome-wide ES cell data we performed ChIP using antibodies against CTCFL and CTCF on selected sites in cells isolated from wild-type testis by elutriation. Results show preferential binding of CTCFL to the Stra8 and Prss50 promoters, and preferential binding of CTCF to Vps18 (Figure 7F), indicating that the differential binding pattern of CTCF and CTCFL observed in ES cells is present in testis as well.

### CTCFL is functionally different from CTCF

**Figure 8:**
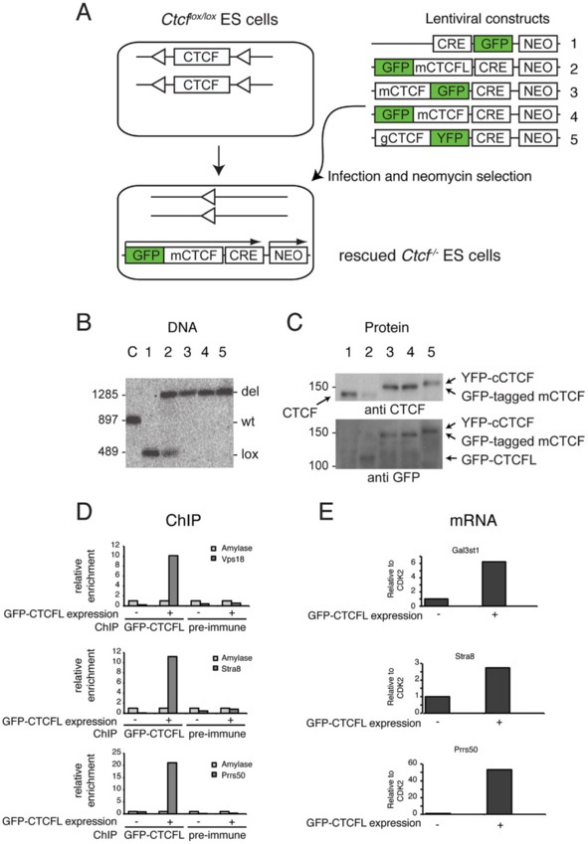
**CTCFL is functionally different from CTCF A** Strategy for the rescue of CTCF-depleted ES cells. Ctcf^lox/lox^ ES cells were infected with lentivirus containing the Cre recombinase and/or fluorescently tagged CTCF(L) proteins. After infection neomycin-resistant colonies were picked and analyzed. *m* = mouse, *g* = chicken. **B** Analysis of Ctcf^lox/lox^ deletion. After infection with CRE-containing constructs, Ctcf^lox/lox^ ES cells were scored for the status of the conditional Ctcf alleles by DNA blot. The position of wild-type (wt), deleted (Ctcf^del^, or del) and conditional (Ctcf^lox^, or lox) loci in control ES cells (**C**), non-treated Ctcf^lox/lox^ ES cells (*1*) and lentivirally transduced Ctcf^lox/lox^ ES cells (*2*–*5*, see panel **A** for numbering of constructs) is indicated. Cells are considered rescued when both conditional CTCF alleles have been deleted. **C** Analysis of CTCF protein expression. Neomycin-resistant colonies were grown and analyzed by Western blot for CTCF (*upper panel*) or GFP (*lower panel*) expression. Note that rescued cells are negative for endogenous CTCF. **D**, **E** GFP-CTCFL is a functional protein. ES cells were transiently transfected and harvested after 1 day. ChIP (DNA, **D**) and RT-PCR (mRNA, **E**) analyses revealed that GFP-CTCFL binds Vps18, Stra8 and Prss50 promoters (**D**) and is able to induce expression of Gal3st1, Stra8 and Prss50 (**E**).

The co-expression of CTCFL and CTCF in late spermatogonia and preleptotene spermatocytes combined with their differential genome-wide binding patterns raises the question whether CTCFL and CTCF are functionally redundant, complementary or antagonistic. To test whether CTCFL can functionally substitute for CTCF, we designed an ES cell rescue assay (Figure 8A). ES cell lines were derived from mice carrying the conditional Ctcf knockout allele (Ctcf^lox/lox^[4]). Ctcf is deleted upon lentivirus-mediated Cre recombination, and these cells fail to form colonies because CTCF-deficient cells (Ctcf^del/del^) do not survive. A rescue of cell death by concurrent introduction of CTCFL would show that the two proteins compensate for each other.

Using this strategy we co-expressed Cre with GFP-tagged mouse CTCF, YFP-tagged chicken CTCF or GFP-tagged mouse CTCFL in Ctcf^lox/lox^ ES cells (Figure 8A). Resulting colonies were analyzed on the DNA level for Cre-mediated CTCF deletion of Ctcf^lox/lox^ into Ctcf^del/del^ (Figure 8B) and on the protein level for expression of endogenous or exogenous protein (Figure 8C). A few surviving colonies transduced with Cre-only were observed (Table 3), but these had not performed the Cre-mediated CTCF deletion completely and thus still expressed endogenous CTCF (Figure 8B, C). However, nearly all colonies transduced with N- or C-terminally tagged mouse CTCF, or with C-terminally tagged chicken CTCF, had deleted endogenous Ctcf^lox/lox^, and expressed fluorescently tagged exogenously introduced protein (Figure 8B, C, Table 3). Thus GFP-tagged mouse CTCF and even chicken CTCF, which is 96% identical at the amino acid level to mouse, can functionally substitute for endogenous CTCF.

**Table 3:** Rescue of CTCF-deficient ES cells by exogenously introduced GFP-CTCF(L)

**Species**	**Construct**	Deletion in ES cells**	Partial deletion in ES cells**	No deletion in ES cells**	**Functional CTCF substitution**
N.a.*	GFP-CRE	0% (0/65)	28% (18/65)	72% (47/65)	No
Mouse	GFPCTCFL-	0% (0/18)	50% (9/18)	50% (9/18)	No
Mouse	CTCFGFP- CRE	83% (19/23)	0% (0/23)	17% (4/23)	Yes
Mouse	GFPCTCF- CRE	95% (40/42)	2% (1/42)	2% (1/42)	Yes
Chicken	CTCFYFP- CRE	90% (37/41)	2% (1/41)	7% (3/41)	Yes

*: Not applicable.

**: Percentage and number of clones (between brackets) in which the conditional CTCF alleles were deleted, partially deleted or not deleted are shown.

Strikingly, rescue experiments with GFP-tagged mouse CTCFL yielded no ES cells in which both endogenous Ctcf^lox/lox^ alleles were deleted and wild-type protein was replaced (Figure 8B, C, Table 3). These data indicate that either CTCFL and CTCF are not interchangeable or that GFP-CTCFL is not a functional protein. To demonstrate that GFP-CTCFL is functional, we transiently transfected the protein into ES cells and examined DNA binding of GFP-CTCFL on selected sites and the induction of expression of testis-specific genes. ChIP experiments showed that GFP-CTCFL binds the three selected sites (Figure 8D) and that Gal3st1, Stra8 and Prss50 expression is induced inES cells expressing this fusion protein (Figure 8E). These data demonstrate that GFP-CTCFL is functional.

## Discussion

We have used a combination of approaches and technologies to unravel the physiological function of the testis-specific paralog of CTCF, called CTCFL or BORIS. We find that CTCFL is only expressed in late spermatogonia and preleptotene spermatocytes, and that CTCFL-deficient mice have defects in spermatogenesis. CTCFL and CTCF are functionally different proteins. CTCFL therefore has a unique role in the adult testis. It has been proposed that CTCFL is involved in genomic imprinting of the *Igf2-H19* locus and other sites [19,24]. However, imprint-related mutations often have embryonic phenotypes [25]. We did not observe this in *Ctcfl*^*del/del*^ mice, and despite their reduced fertility *Ctcfl*^*del/del*^ mice could be bred through multiple generations. Furthermore, we have not been able to detect DNA methylation aberrations in specific loci in *Ctcfl*^*del/del*^ mutant mice and in CTCFL-over-expressing cells (data not shown). This makes a role for CTCFL in DNA methylation-dependent genomic imprinting unlikely. The combined microarray data from CTCFL-deficient testis and CTCFL-expressing ES cells, and the preference of CTCFL for promoters instead suggest a function as a transcriptional regulator, required for the proper expression of a subset of male germ cell genes.

The most prominent CTCFL-binding sites in ES cells are on the promoters of the testis-specific Stra8 and Prss50 genes. The expression of these genes, and of Gal3st1, is upregulated in ES cells expressing CTCFL. Conversely, expression of Prss50 and Gal3st1 is downregulated in germ cells lacking CTCFL, at all ages examined, whereas Stra8 expression is affected at some but not all ages (data not shown). We speculate that the combined transcriptional deregulation of genes causes the testicular degeneration and reduction in fertility in Ctcfl knockout mice. Note that the expression of these genes is not completely hampered, which explains why the testicular phenotype in the knockouts is milder than the fully sterile phenotype described, for example, for STRA8- and GAL3ST1-deficient mice [29,30,35].

The phenotype of the *Ctcfl*^*del/del*^ mice reported here only partly matches a recent report on another strain of CTCFL-deficient mice, in which exons 1 to 8 of Ctcfl were also deleted [26]. For example, the effect of a Ctcfl deletion on the average testicular size and on Gal3st1 and Prss50 expression is similar. However, our analysis also reveals a reduction in fertility in the *Ctcfl*^*del/del*^ mice not noted previously [26]. In addition, the fact that some *Ctcfl*^*del/del*^ mice have normal testis size and others have a combination of normal and abnormal seminiferous tubules was also not described. This is relevant, as this incomplete penetrance of the Ctcfl phenotype, even within a single testis, suggests that a stochastic mechanism determines whether CTCFL-deficient tubules degenerate or not. Finally, CTCFL was proposed to be present in round spermatids and to function during meiosis based on mRNA expression data [26]. By contrast, our data show that CTCFL is expressed earlier, just prior to the onset of meiosis, and we conclude that CTCFL protein expression precedes the developmental germ cell stages that show the major phenotypes in Ctcfl knockout mice. We propose that in the absence of CTCFL, epigenetic marks controlled by this protein gradually break down in a stochastic manner. Spermatogonia and primary spermatocytes exist in syncitia, in which each cell is connected with the other cells at the same step of development via intercellular bridges. Only in syncytia where the expression of CTCFL-controlled genes has been affected beyond a specific threshold will degeneration become apparent.

Neither CTCFL nor CTCF is saturating all consensus-binding sites present in the genome, and thus the DNA sequence is not the sole determinant of CTCF(L) binding. DNA methylation and hydroxymethylation are not a decisive aspect, as comparisons of DNA (hydroxy)methylation data sets to our CTCF(L)-binding sites does not provide an explanation for why CTCFL and CTCF occupy different binding sites (data not shown) [47]. Instead, the data suggest that binding of CTCFL and of the “master weaver” CTCF is specified by nucleosome occupancy and composition. We find that CTCFL prefers CTCF consensus sites in promoters that are embedded in regions that appear to be nucleosome-free. By contrast, CTCF is enriched on distinct sites, which are devoid of histone H3 on the binding site itself, but which are surrounded by ordered, or “phased,” nucleosomes. This preference of CTCF has already been described [11-13].

It has recently been shown that unstable nucleosomes are lost when histones are prepared with conventional conditions; thus, regions containing these histones appear as nucleosome-free in the analysis, but are in reality not free [45]. Nucleosomes containing the variant histone H3.3 are quite unstable, and those containing both H3.3 and H2A.Z even less [44]. Since we find a correlation between CTCFL binding and H3.3 occupancy in ES cells, H3.3 and H3.3/H2A.Z might be determinant factors able to attract CTCFL and evict CTCF. It is important to realize that in ES cells H3.3-enriched genomic regions do not require CTCFL to be set up, yet the protein prefers such areas after its induction. A similar situation may exist in testis, i.e., specific H3.3/H2A.Z-containing regions might be set up during early phases of spermatogenesis; upon its expression, CTCFL “lands” on these regions, possibly evicting CTCF from some promoters. Notably, during male meiosis, and thus subsequent to CTCFL expression, H3.3 is incorporated into unsynapsed chromatin, which is transcriptionally inactive [48]. The function of CTCFL might be to ensure the expression status of genes by distinguishing specific promoter-associated H3.3 domains from whole chromosome domains that also contain H3.3. Through its interaction with SET1A [49], CTCFL might enhance H3K4 trimethylation at a subset of its binding sites.

The cohesin complex has a role in chromosome segregation, DNA-damage repair and gene regulation [50]. Although cohesin does not have a typical DNA-binding motif, it was shown to bind primarily to CTCF consensus sites [16,17,51]. Moreover, the SA2 subunit of cohesin directly interacts with the C-terminus of CTCF [52].

Cohesin’s role in gene regulation therefore seems tied to that of CTCF. Recent studies revealed that also in ES cells cohesin binding largely overlaps with that of CTCF; however, there are ~2,000 cohesin sites with a CTCF motif that do not bind CTCF, while ~270 other cohesin sites do not have a CTCF consensus site [10]. Our data suggest that CTCFL binds these ~2,000 cohesin sites in CTCFL-GFP-V5-expressing ES cells.

However, in normal ES cells CTCFL is not expressed, raising the questions how a specific nucleosome composition and occupancy can be built around CTCF consensus sites that appear not to be occupied by CTCF, and how cohesin can stably bind these very same sites. We hypothesize that these sites might be bound by a modified form of CTCF, such as poly(ADP-ribosyl)ated CTCF [53]. This protein would not be able to bind DNA tightly and could be replaced very efficiently by CTCFL. Perhaps another molecular function of CTCFL in the testes is to interfere with and/or change the dynamics of CTCF and cohesin-mediated chromatin looping.

We observed competition between CTCF and CTCFL in ES cells, but only on a small subset of all CTCF-binding sites. Nucleosome occupancy and composition, CTCF(L) expression levels and posttranslational modifications on CTCF(L) could all determine whether competition between the proteins occurs on a given site. Our data reveal that CTCF and CTCFL co-localize within the nuclei of late spermatogonia and preleptotene spermatocytes, and the proteins might therefore also compete in vivo. ChIP experiments in testis extracts indeed reveal preferential binding of CTCFL at the Stra8 and Prss50 promoters and exclusive binding of CTCF to the Vps18 site. These data are consistent with binding profiles in ES cells. If competition on the Stra8 and Prss50 genes does occur in vivo, then CTCFL could be a gene activator by preventing the binding of CTCF. In Ctcfl knockout mice binding of CTCF to these genes might actually diminish their expression. However, CTCF is ubiquitously expressed in the testis, whereas CTCFL is only transiently present in spermatogonia and preleptotene germ cells. One would expect to see significant binding of CTCF to the Stra8 and Prss50 sites in the testicular extracts that we used, since most cells in these extracts contain CTCF and not CTCFL. The questions why CTCF is not highly enriched on the Stra8 and Prss50 promoters in testis, and whether these proteins compete in vivo can only be answered once there are tools available to isolate CTCFL-positive and -negative cell populations from testis so that genome-wide analyses can be performed on purified testicular fractions.

In human germ cell tumors, CTCFL is specifically upregulated in spermatocytic seminomas, which are benign testicular tumors originating from a spermatogonium or primary spermatocyte [54]. This fits with our observed cellular localization of CTCFL and could potentially point to an oncogenic role for CTCFL in these tumors. In fact, CTCFL belongs to the group of cancer testis antigens (CTAs), genes that are normally expressed in testis yet aberrantly expressed in a variety of cancers. One model holds that competition between CTCF and CTCFL plays a role in tumorigenesis, i.e., aberrant CTCFL expression would displace CTCF, and affect DNA methylation and the expression of other CTAs, including the NY-ESO-1 and MAGE-A1 genes [22,23], and even other important genes, such as the TERT gene, which encodes telomerase [55]. However, while there might be a relationship between DNA demethylation and the expression of CTAs [56], recent reports have shown that expression of CTCFL alone is not sufficient to induce expression of CTAs [27,57]. Furthermore, our data in CTCFL- deficient testis indicate that, if anything, CTCFL represses the Tert gene instead of activating it. To address a potential role of CTCFL in cancer, a correlation analysis of CTCFL binding, nucleosome occupancy and composition, and CTA expression in different types of cancers might be more revealing.

## Conclusions

The three-dimensional folding of the eukaryotic genome serves to compact DNA while allowing gene expression. CTCF has been termed the “master weaver” of the genome, since this protein is a key coordinator of chromatin loop formation. In this study we have analyzed the physiological function and DNA-binding profile of CTCFL, a protein that is highly similar to CTCF but that is only expressed in the male germ line. Using a combination of cell biological, biochemical and bioinformatics approaches, we show that CTCF and CTCFL are functionally different proteins that bind to similar sites in the genome, but whose binding does not overlap completely. Our data suggest that nucleosome composition specifies the genome-wide binding of both CTCFL and CTCF. We show that CTCFL is only transiently expressed, in spermatogonia and preleptotene spermatocytes, prior to male meiosis. We propose that during its expression CTCFL occupies a subset of promoters and thereby maintains the expression of selected male germ cell genes.

## Methods

### RACE PCR and RNase protection assay

Human CTCFL was shown to consist of 23 isoforms with variations in N- and C-termini and zinc finger modules with different DNA-binding and transcriptional characteristics [58]. To analyze the genomic organization of the murine Ctcfl gene, we cloned the 5’ end of the Ctcfl cDNA by a rapid amplification of cDNA ends-polymerase chain reaction (RACE-PCR) procedure, using first choice RACE ready testicular cDNA from Balb/c mice (Ambion) and nested oligos (see Table 4 for sequence). Compared to the published murine Ctcfl sequence [19], the RACE-PCR-derived first Ctcfl exon is smaller and lacks an upstream ATG, and it is preceded by an intron of 489 bp and an additional exon of 130 bp (see also panel 1C). The sequence of the Ctcfl 5’end product has been submitted to Genbank (accession no.: EU154995). Our cDNA structure matches the HAVANA/VEGA curated sequences in Ensembl, Build 36 [59].

**Table 4:** Oligos

	**Oligos used for RACE PCR**		
**Name**	**Forward**	**Backward**
RACE	GGACACTCGTATTTGGGCACATTC	CACAGGGAGCACTTGAAGGGCTTC
	**Oligos used in real-time PCR for ChIP**		
**Name**	**Forward**	**Backward**	
Gal3st1	TCCTGGGTGAGGTCAGGAAG	GGAACTCCGAGTAGCTTCAATG	
Stra8	TCCTAGAGAAGGGGGTGTTACC	AGCTGACCACCACACGTTTTC	
Prss50	AGAGGAGGGTAGGGGTATCGAC	TCGCCTCAGCTAATTTCTAAGC	
Vps18	CTGCTGCAGTTCCTCATGTTG	GTGTGACAGATGGAGGAGCAC	
Chr10	AAGGTTGGTAGCTCTGCTTGGACTGCTCG	AATGTCACAAGCAAAGAAAAGCACGCAAAT	
Amylase	AATTCTCCTTGTACGGCTTCGTG	TAGCAATGATGTGCACAGCTGAA	
	**Oligos used in real-time PCR for RNA expression**		
**Name**	**Forward**	**Backward**	
Clgn	TGTCTTCCTTACTCTTCTCTTCCG	GAAGCCAGGTGAAGCTGAGGTC	
Ctcf	CCACCTGCCAAGAAGAGAAGG	GCACCTGTATTCTGATCTTCGAC	
Tcfl5	ACGAGATAGGAGGCGCAGAATC	GTTGTTGCTTTATCTGTCTCCG	
Gal3st1testis-specific form	GCTACTCGGAGTTCCGGAAA	GACTTGCAGGGCTTCTTTGG	
Gal3st1	ACTGTATCCCAACATGGCCTTC	ATATCTCGCCGAGGTTGACAC	
Stra8	GGCAGTTTACTCCCAGTCTGATA	CAACTTATCCAGGCTTTCTTCCT	
Prss50	GACAGTTCTCTCTGCACTGTGAC	CACATTTCTTGCTGTTCAGGATA	
Ctcfl	GCTCTGGCTGTGCACCTTACG	CCCACTGTGCCACCATCATC	
Ccna1	GAGTATGCAGAGGAGATTCATCG	TCATGTAGTGAGCCTTGGGTCTG	
lpcat2	AGCACCCAGTGAGGAAGAGA	TTCGTAGGTGTGATCCGTCA	
itfg3	ACGAGGTGTCTTCTGCCTGT	GTTCCCACTAAAGCTGCTGG	
dio2	TGCAGATCCTGCCAGTCTTT	CACACTGGAATTGGGAGCAT	
hgf	GATGAGTGTGCCAACAGGTG	GGTCAAATTCATGGCCAAAC	
akr1c18	CCAGGCCATTCTAAGCAAGA	TCAGGGAATTTTCCAAGCTG	
	**Oligos used in EMSA**		
**Name**	**Forward**	**Backward**	
Stra8	GGATCTGTGCTGTGTGTCCTCCTCGACTCCT	CCTCTAGGAGTCGCAAGTGACCCACACATGCATGC	
	AGAGCATGCATGTGTGGGTCACTTGCGACTC		
	CTAGAGGA	TCTAGGAGTCGAGGAGGACACACAGCACAGATCCT	
Prss50	ATCTAGGGGGCGCCACGCAGGCTGGGCACC	CCACAATGGCGCCCTCCATCGGGCGCCTCATGGT	
	AGCGCACCATGAGGCGCCCGATGGAGGGCG	GCGCTGGTGCCCAGCCTGCGTGGCGCCCCCTAGATG	
	CCATTGTGGA		
	**Oligos used for generating transgenic mice and cells**		
**Name**			
Oligo 1	5-TCTTTTTCCATCAGGGGTCGTCAC-3		
Oligo 2	5-GAGAAGCGCGATCACATGGTCCTG-3		
Oligo 3	5-GCACCGTTTGCAGGGTCAGGATC-3		
Oligo 4	5-TCCAAATCACAGCGCCACCTACAG-3		
Oligo 5	5-GGTTCTTAGAGATAGGGTTTCTCTG-3		
Oligo 6	5-GGTGTTCTGCTGGTAGTGGTC-3		
Oligo 7	5-CGGCATCAGAGCAGCCGATTG-3		
Oligo 8	5-GTTATGATCTGGGTATCGTCCACTG-3		

RNase protection assay (RPA) was performed according to the manufacturer’s instructions (RPAII, Ambion). For Ctcfl the RPA was performed on poly A + purified RNA with probes spanning Ctcfl exons 8 and 9 (protecting 146 bp), spanning bp 1–220 of the Ctcfl race PCR product (up to oligo 3, see figure S1C) or, alternatively, spanning bp131-220 (protecting 89 bp), to detect the existence of another start site [19]. For Ctcf the RPA was performed on total RNA with a probe protecting 99 bp of Ctcf exon 2. The RPA with the 5’end RACE confirms that *Ctcfl* mRNA mRNA contains the additional upstream exon as identified by the RACE PCR. We found no evidence for alternative splicing in murine Ctcfl.

### Mouse models

To generate the Ctcfl and Ctcf knockin alleles we inserted a Gfp-encoding cDNA, followed by a Loxp-flanked neomycin selection cassette, in the Ctcfl and Ctcf exons, respectively, that contain the ATG start codons. Insertion of GFP immediately downstream of the translational start sites yielded *Ctcfl*^*gfp*-neo^ and Ctcf^gfp-neo^ ES cells. Homology arms were generated by cloning from the RPCI21 129 PAC library (Geneservice). Constructs were sequenced, electroporated into isogenic ES cells (129/IB10) and neomycin- (or, later on, puromycin-) selected, analyzed by Southern blot and PCR, and injected into C57/Bl6 blastocysts.

Mice generated from *Ctcfl*^*gfp*-neo^ and Ctcf^gfp-neo^ ES cells were crossed to transgenic mice expressing Cre to delete the LoxP-flanked neomycin cassette. This yielded *Ctcfl*^*gfp*^ mice in which the GFP is fused in frame to CTCFL and Ctcf^gfp^ mice where GFP is fused to CTCF. These mice are phenotypically normal and fertile (data not shown).

The *Ctcfl*^*gfp*-neo^ ES cells were retargeted with a LoxP-flanked puromycin cassette downstream of exon 8. Mice were generated using the *Ctcfl*^*gfp*-neo-puro^ ES cells. The *Ctcfl*^*del*^ mice were subsequently generated by crossing *Ctcfl*^*gfp*-neo-puro^ mice to Cre-expressing mice. This resulted in the in vivo deletion of Ctcfl exons 1–8 and both selection cassettes as these were in between the LoxP sites.

Mice were maintained on a C57/Bl6 background at the Erasmus MC animal care facility under specific pathogen-free conditions. Animal experiments were reviewed and approved by the Erasmus University committee of animal experiments.

### Cell culture, transfection and infection

The Ctcf^lox/lox^ ES cells were isolated de novo from CTCF conditional mice [4] and grown on plastic in the presence of LIF. Lentiviral constructs were generated with Ctcf and Ctcfl cDNAs driven from a CAG promoter (CMV early enhancer/chicken β actin), followed by an IRES sequence and the Cre recombinase. Expression of a neomycin selection cassette was driven by a PGK promoter. Lentivirus particles were produced as described (Addgene). ES cells were infected in suspension for 4 h, plated and selected with G418 the next day. Clones were analyzed by Southern blot for the status of the Ctcf^lox/lox^ conditional allele [4] and by Western blot using GFP (Abcam 32146) or CTCF antibody (BD Bioscience).

For the inducible CTCFL expressing ES cells, ROSA26-rtTA ES cells were Lipofectamine transfected (Invitrogen) with a TRE-mCTCFL-V5-GFP-neomycin construct and selected with G418. Clones were analyzed for the induction and expression of CTCFL-V5-GFP by flow cytometry for GFP (FACSAria, BD Biosciences), and by Western blot and immunofluorescence using rat monoclonal anti-CTCFL antibodies raised against mouse CTCFL (AA 1–113 and AA 569–635) and V5 antibody (Sigma, V8012). Transient transfections of mCTCFL-V5-GFP and of GFP-mCTCFL in ES cells were done with Lipofectamine 2000 (Invitrogen).

### Chromatin immunoprecipitation (ChIP)

\ChIP was performed as described [14] or according to the Magnify system procedure (Invitrogen). Briefly, preparation of cross-linked chromatin (2 × 10^7^ cells treated with 1% formaldehyde for 10 min at room temperature), sonication of chromatin to yield fragments up to 800 bp, and immunoprecipitation with V5-Agarose beads (Sigma, A7345) or with polyclonal CTCF(L) antibodies [4] were performed as described in the Upstate protocol (http://www.upstate.com). Ct values from real-time PCR were normalized to input measurements, and enrichment was calculated relative to the Amylase gene. For oligos used, see Table 4. ChIP was performed on nuclei derived from induced or transiently transfected ES cells (see above) or from seminiferous tubules in which multiple testicular cell populations were first dissociated by enzymatic digestion of seminiferous tubules and subsequently isolated by elutriation.

### ChIP-sequencing and analysis

For ChIP-sequencing a DNA library was prepared from the ChIPped DNA according to the Illumina protocol (http://www.illumina.com). Briefly, 10 ng of end-repaired DNA was ligated to adapters, size selected on gel (200 ± 25-bp range) and PCR amplified using Phusion polymerase as follows: 30 s at 98 °C, 18 cycles of (10 s at 98 °C, 30 s at 65 °C, 30 s at 72 °C) and 5 min at 72 °C final extension. Cluster generation was performed using the Illumina Cluster Reagents preparation, and the library was sequenced on the Illumina Genome Analyzer IIx platform to generate 36-bp reads. Images were recorded and analyzed by the Illumina Genome Analyzer Pipeline (GAP) and processed using the IPAR (Integrated Primary Analysis Reporting Software) and the GAP. The resultant sequences were mapped against NCBI Build 37.1 of the mouse genome using the ELAND alignment software (Illumina).

Published data sets generated for mouse ES cells were downloaded from NCBI’s gene expression omnibus (GEO). We used the following data sets: H3: GSM587479, CTCF: GSM288351 [7], Smc1: GSM560341 and GSM560342 [8], H3K4me3: GSM594581 [42], PolIIser5p: GSM515662 [43] and H3.3-HA: GSM423355 [46]. Reads were converted to the fastq format and aligned to a precompiled mm9 reference index with BOWTIE [60]. In case multiple sequencing lanes were available, fastq files were merged before alignment. Unambiguously mapped and unique reads were kept for subsequent generation of binding profiles and calling of peaks using MACS with an fdr < 0.05 [61]. Downstream analysis was done in R/BioConductor (http://www.bioconductor.org), partly according to published strategies [62].

For comparative ChIP-Seq analysis mapped reads were transformed to continuous binding profiles. Those were used to collect data in 4-kb windows spanning CTCF and CTCFL binding sites. The binding sites were grouped into three classes based on intersection analysis: sites bound by CTCF only, CTCFL only, or both CTCF and CTCFL. The binding data were binned across binding sites in 50-bp windows, and the mean was calculated at each position in order to generate cumulative binding profiles. Alternatively the complete data were plotted in heatmaps. The identified CTCFL-binding motif was used to scan the mm9 genome using the Patser tool [63] and plotted as a heatmap after the motif data had been binned, as explained for the binding profiles.

### RNA analyses

Expression analyses by real-time PCR were performed as follows: total RNA was isolated with RNA-Bee (Tel-Test Inc.). RNA was reverse transcribed (RT) with a combination of random and oligo-dT primers by Superscript reverse transcriptase (Invitrogen), and real-time RT-PCR was performed with a Sybrgreen platform on a Bio-Rad CFX Cycler. For oligos used, see Supplemental Information.

For testis and ES cell microarray analysis, the purity and quality of the isolated RNA were assessed by RNA 6000 Nano assay on a 2100 Bioanalyzer (Agilent Technologies). Then 5 ug testes RNA was used for the production of cRNA. Labeled cRNA was hybridized to the GeneChip Mouse Genome 430 2.0 array oligonucleotide microarray (Affymetrix) according to manufacturer’s recommendations; 300 ng ES cell RNA was used for production of end-labeled biotinylated ssDNA. Labeled ssDNA was hybridized to the Mouse Gene 1.0ST array (Affymetrix) according to manufacturer’s recommendations. Measured intensity values were analyzed using the Gene Expression Console (Affymetrix) and normalized by quantile normalization.

Scanned microarray data were processed using R/Bioconductor using standard procedures. Normalization and background correction were done by RMA. Differentially expressed genes were determined using the limma package within R [64]. For visualization the mean expression was determined across the heterozygous samples, which was then subtracted from the expression levels for the individual samples. For the analysis of the association between gene expression and CTCFL/CTCF binding, RefSeq genes were downloaded from the UCSC genome browser homepage. For each gene represented on the MoGene 1.0 ST array, the nearest CTCF or CTCFL site was calculated. Genes with a binding site within an interval from −2 kb to +1 kb around the transcriptional start sites were determined as bound. Log2-transformed expression values derived from Affymetrix analysis of mES cells was then plotted for the identified genes.

All Chip-seq and Microarray datasets are available at NCBI Gene Expression Omnibus (http://www.ncbi.nlm.nih.gov/geo/) under accessions: GSE34091, GSE34092, GSE34093 and GSE34094.

### Electrophoretic mobility shift analysis (EMSA) or band shift analysis

Nuclear extracts were obtained from mock-transfected HEK 293 T cells and HEK 293 T transfected with pEGFP, pEGFP-mCTCF or pEGFP-mCTCFL. After 24 h, cells were harvested, washed with cold PBS, resuspended in buffer 1 [10 mM HEPES; 10 mM KCl; 0.25 mM EDTA pH 8, 0.125 M EGTA-K pH 8, 0.5 mM Spermidin, 0.1%, NP40, 1 mM DTT, protease inhibitor cocktail set I (Calbiochem)] and incubated for 10 min on ice. Cells were then centrifuged 5 min at 1,500 rpm. The supernatant was removed, and the pellet was resuspended in 50 μl of buffer 2 [20 mM HEPES; 0.4 M NaCl; 0.25 mM EDTA, 1.5 mM MgCl2, 0.5 mM DTT, protease inhibitor cocktail set I (Calbiochem)] and incubated 1 h at 4 °C. Samples were centrifuged for 30 min at 15,000 rpm, and the supernatant (nuclear extract) was frozen at −70 °C until used.

Radiolabeled probes were generated by PCR of genomic DNA (for oligos used, see Table 4). In all cases the PCR was performed in a final volume of 50 μl containing 3 μl of [α-32P]dCTP (Hartman Analytic), 20 ng of genomic DNA (from K562 cells), 0.2 mM each dNTP, 0.5 μM of each primer and 1U of DFS-Taq DNA polymerase (BIORON). The PCR fragments were purified using the Wizard SV Gel and PCR CleanUp System (Promega).

The EMSA reaction was performed by mixing 10 μg of the nuclear extract with 6 μl of EMSA buffer (1.5 μg of poli-dIdC, 20 mM HEPES pH 7.5, 50 mM KCl, 5% glycerol, 0.175 mM EDTA) in a volume of 19 μl. Mixtures were pre-incubated at 25 °C for 10 min. Then 162 μl of the radiolabeled probe was added to each condition, and the resulting mixture was incubated for 30 additional min at 25 °C. For competition, 10 μg of CTCF nuclear extract, followed by increasing amounts of competing extracts, was added to the binding reaction. Then, the mixtures were pre-incubated as previously described. For supershift experiments, 1 μl of anti-CTCF mouse monoclonal antibody (BD Biosciences) or 1 μl of anti-actin (Santa Cruz, sc-1616), used as a non-specific antibody, was added to the binding reaction prior to the radiolabeled probe. Complexes were analyzed by electrophoresis on a 4% polyacrylamide gel at 160 V for 2 h with 0.5×Tris-borate-EDTA buffer. Gels were fixed using 10% acetic acid for 10 min and then dried for 30 min using a Gel Dryer (Bio-Rad). Radioactive complexes were revealed using a Molecular Imager Fx (Bio-Rad).

### Pathological analysis of ctcfl knockout mice

Testis weight was determined immediately after dissection. Weights were measured within the tunica albuginea, excluding the cauda epididymis. Sperm analysis and counts were performed as described [33]. The epididymis was dissected and transferred into a small conical glass grinder, and homogenized by hand. The total number of sperm present in the epididymis was counted using a Neubauer hemocytometer and a phase contrast microscope (magnification 400×). At least 200 sperm in two different samples were counted. Fertility of mice was determined by breeding the mice to multiple mates and scoring the number of offspring.

### Immunofluorescence and immunohistochemistry techniques

For BrdU incorporation, mice were injected intraperitoneally with 1.2 μg BrdU. One hour after injection, testes were dissected, fixed in 4% paraformaldehyde or Bouin, paraffin embedded and sectioned. For H/E staining, Bouin or PFA fixed testes were fixed overnight at 4 °C, washed and dehydrated using ethanol and xylene, and embedded in paraffin. Sections of 10 μm were cut, mounted on SuperFrost Plus slides (Menzel-gläser), rehydrated and stained with H/E.

For immunofluorescence analyses, Bouin or PFA fixed and paraffin embedded testes were sectioned, treated with the microwave (three times for 5 min, 750 W) in 10 mM NaCitrate buffer (pH 6.0) to expose antigens and stained using standard procedures. Antibodies used: Rat monoclonal anti-CTCFL (see abobe), PRSS50 (Abcam 49405) and STRA8 (Abcam 49405).

### Live imaging in seminiferous tubules

Imaging of testis tubules was performed as described [33]. Briefly, testis were injected through the rete testis with Hoechst 33342 and Trypan blue (Sigma) in 3–5 μl of PBS, 1 h prior to testis dissection, to allow spreading of the vital DNA stain throughout the adluminal compartment of the testis tubules and uptake by nuclei on the adluminal side of the Sertoli cell barrier. Trypan blue served as a marker for injected tubules. Individual seminiferous tubules were isolated from testes using a collagenase and hyaluronidase method, and Trypan blue positive tubules were transferred into a drop of PBS + with 0.2% BSA in a live-cell chamber overlaid with PBS-saturated mineral oil. The testis tubules were examined at 33 °C, using a Zeiss LSM510NLO confocal/multiphoton setup, to allow simultaneous acquisition of phase-contrast, GFP and Hoechst images.

## Abbreviations

BrdU, bromodeoxyuridine; ChIP, chromatin immunoprecipitation; DAB, diaminobenzidine; dCTP, deoxycytidine triphosphate; dNTP, deoxynucleoside triphosphates; DTT, dithiothreitol; EDTA, ethylenediaminetetraacetic acid; EMSA, electrophoretic mobility shift analysis; H/E, hematoxylin/eosin; HEPES, 4-(2-hydroxyethyl)-1-piperazineethanesulfonic acid; PCR, polymerase chain reaction; PBS, phosphate-buffered saline; PFA, paraformaldehyde; RMA, robust multi-array average; RPA, RNase protection analysis; TSS, transcription start site.

## Competing interests

The authors declare that they have no competing interests.

## Authors' contributions

FS, WS, MB, HH, SD, PB, VF, MR-G, SvdN, LC, MvdR, JCB, WvIJ, BL, MDD, RR, and NG contributed to acquisition of data. FS, WS, MB, SD, PB, MR-G, WvIJ, AG, MDD, RR, FG, and NG designed the experiments, and analyzed and interpreted the data. FS, WS, MB, RR, and NG drafted the manuscript which wasapproved by all authors.

## Supplementary Material

Additional file 1 **Movie S1.** Live imaging of GFP-CTCFL. Ex vivo confocal imaging of a live seminiferous tubule derived from a *Ctcfl*^*gfp*^ knockin mouse. Images were acquired throughout tubules using a combined multiphoton (Hoechst) and confocal laser (GFP) scanning microscope setup. Images were assembled for 3D reconstruction afterwards. The GFP-CTCFL fusion protein is shown in green. The DNA stain Hoecht, which was injected at the adluminal site of the testis tubule, is shown in red. Hoechst-positive cells represent Sertoli cells, leptotene stage IX and later stage spermatocytes, and spermatids, all of which are negative for GFP-CTCFL. Notice the presence of the GFP-CTCFL-positive cells on one side (basal lamina) of the tubule. Click here for file
